# Autonomic entrainment to expressive musical phrase structures

**DOI:** 10.1093/ehjimp/qyag149

**Published:** 2026-08-25

**Authors:** Natalia Cotic, Vanessa C Pope, Mateusz Soliński, Pier D Lambiase, Elaine Chew

**Affiliations:** School of Biomedical Engineering & Imaging Sciences, Faculty of Life Sciences & Medicine, King's College London, Strand, London WC2R 2LS, UK; School of Biomedical Engineering & Imaging Sciences, Faculty of Life Sciences & Medicine, King's College London, Strand, London WC2R 2LS, UK; Department of Engineering, Faculty of Natural, Mathematical & Engineering Sciences, King's College London, Strand, London WC2R 2LS, UK; School of Biomedical Engineering & Imaging Sciences, Faculty of Life Sciences & Medicine, King's College London, Strand, London WC2R 2LS, UK; Department of Engineering, Faculty of Natural, Mathematical & Engineering Sciences, King's College London, Strand, London WC2R 2LS, UK; Institute of Cardiovascular Science, University College London and Barts Heart Centre, West Smithfield, London EC1A 7BE, UK; School of Biomedical Engineering & Imaging Sciences, Faculty of Life Sciences & Medicine, King's College London, Strand, London WC2R 2LS, UK; Department of Engineering, Faculty of Natural, Mathematical & Engineering Sciences, King's College London, Strand, London WC2R 2LS, UK

**Keywords:** entrainment, autonomic, coherence, phrase arc boundaries, prosody

## Abstract

**Aims:**

Music reliably influences autonomic physiology, yet the mechanisms linking expressive music structures to cardiorespiratory responses remain poorly understood. This study investigated how phrase-level organization in loudness and tempo relates to autonomic entrainment during music listening.

**Methods and Results:**

We analysed multimodal physiologic recordings [electrocardiogram(ECG)-derived R-to-R intervals, respiration, continuous blood pressure (BP)] from 126 participants in the HeartFM study, each of whom listened to nine expressive solo-piano tracks drawn from a set of 30 (8 original + 22 feature-modulated versions) rendered on a reproducing piano. Music phrase structures were extracted using a Bayesian arc-detection algorithm, generating beat-level boundary profiles for loudness and tempo. Physiologic signals were converted into smooth envelopes and resampled onto the music-beat grid. Entrainment strength was quantified using Earth Mover’s Distance (EMD) between boundary profiles and physiologic envelopes, and evaluated against surrogate distributions. Participant-specific surrogate testing showed that original-vs.-surrogate separation was heterogeneous across physiological signals, with the strongest trend observed for tempo–BP waveform pairings. In contrast, loudness phrases showed consistently lower EMD than tempo phrases across most tracks. Phrase-arc summary statistics (mean and variability of arc length) and music-feature similarity provided measures of predictability. These metrics correlated with entrainment strength; more regular phrase structures were associated with greater synchronization.

**Conclusion:**

Autonomic activity can be mapped to expressive phrase structures, with loudness showing stronger morphology-based alignment than tempo. Predictable phrasing and cross-feature congruence led to greater synchronisation. These findings characterize music–physiology coupling as a structure-dependent, quantifiable phenomenon, providing a mechanistic basis for feature-specific selection of music for eliciting autonomic responses.

## Graphical Abstract

**Graphical Abstract:**
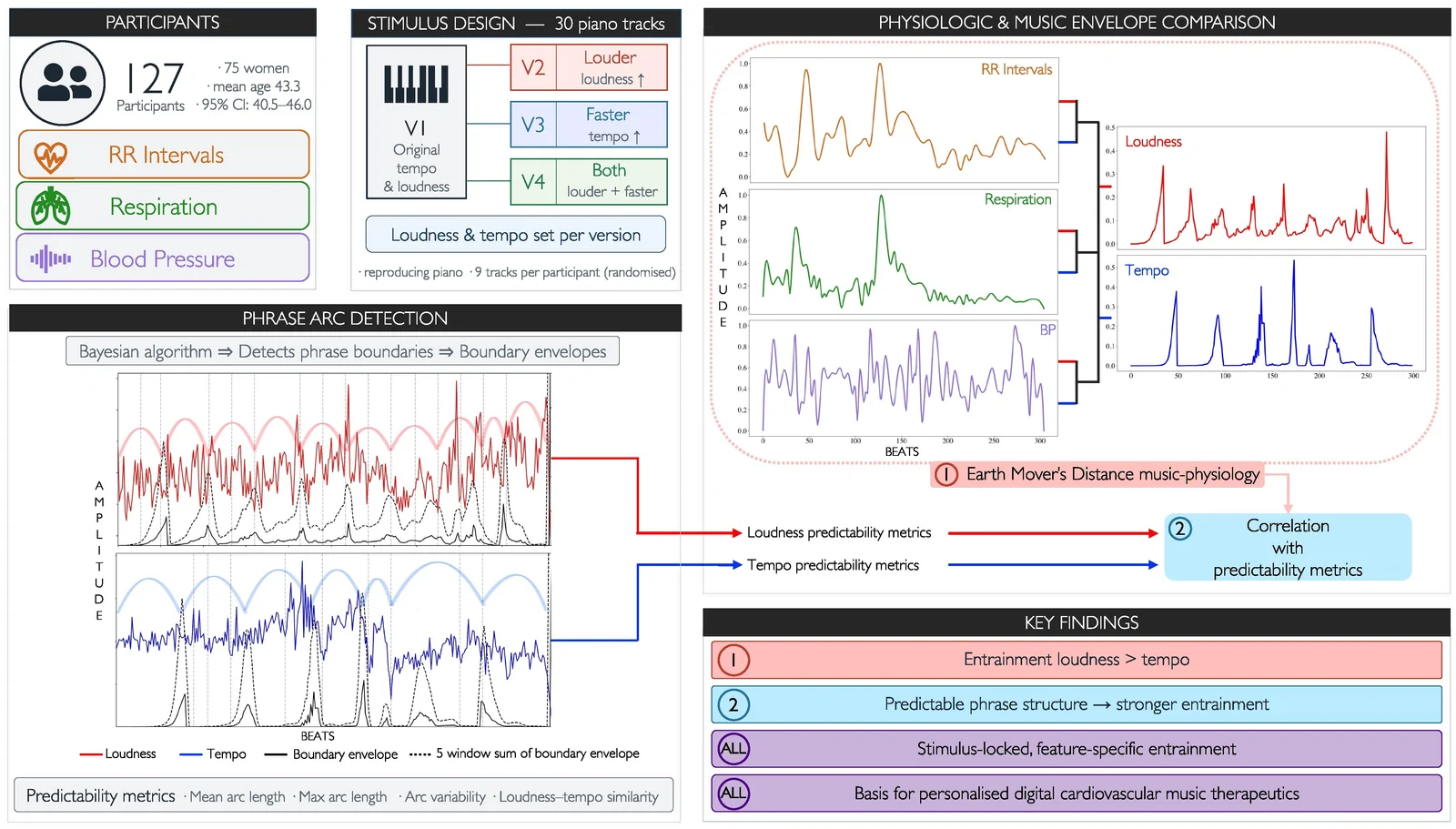

## Introduction

Cardiovascular disease (CVD) remains the leading global cause of death,^1^ prompting interest in non-pharmacological strategies that can complement clinical care. Music-based interventions have emerged as a promising approach for modulating autonomic nervous system function, with reported effects on heart rate, blood pressure (BP), respiration, and related physiologic rhythms.^2–5^ However, the mechanisms through which music stimuli shape autonomic responses remain only partially understood.^6–14^ A key limitation is that prior work often focuses in depth on either physiology or music structure, rather than analysing both at comparable resolution. To develop mechanistically informed music-based interventions, we need to understand not only whether music affects physiology on average but also how specific musical features relate to individual physiological dynamics during extended listening.

A compelling framework for music’s impact on physiology is entrainment,^15^ where internal biological rhythms align with temporal patterns of external stimuli. Entrainment has been observed in response to music rhythm and structure,^16,17^ but further work is needed to identify which music features most strongly support synchronization, and under what conditions. Expression in music often unfolds in arc-like patterns: beat-level variations in loudness and tempo shape perceived phrases and emotion contours.^18–21^ These phrase arcs are believed to guide attention and engagement and may therefore be key drivers of entrainment.^22–24^ Recent computational approaches can identify phrase structure automatically using probabilistic segmentation,^25–29^ enabling direct comparison between phrase-level music structure organization and continuous physiologic responses.

A central challenge is that music features are inter-dependent. Studies investigating tempo and loudness often use heterogeneous stimuli—ranging from metronome clicks to commercial recordings, or simply labelling genres as ‘fast’’ or ‘slow’—confounding tempo and loudness with timbre, instrumentation, lyrics, style, and cultural associations. At the other extreme, reducing music to minimal components removes confounds but can also take away the expressive and structural cues that shape how listeners respond. Expressive features such as tempo and loudness interact to generate expectation and affect; the physiologic impact of music may reflect joint feature dynamics rather than isolated parameters.

In this study, we address these issues using controlled yet expressive solo Western classical piano performances rendered via a reproducing instrument. Tempo and loudness were systematically transposed to create versions of 8 original performances from the Bösendorfer Legendary Artists collection. Details of the recordings can be found in Supplementary Materials  *S1*. We analyse data from the HeartFM protocol,^30^ in which each participant listened to a full playlist while their multimodal physiologic parameters (ECG, respiration, and BP) were recorded continuously. We quantify entrainment between physiologic envelopes and automatically detected music phrase boundaries (for loudness and tempo) and validate findings using within-participant surrogate data to test whether alignment is specific to the music being heard rather than reflecting generic session dynamics. Finally, we examine whether predictability of phrase structure—operationalized through arc summary statistics—relates to entrainment strength, consistent with theories linking predictive engagement to physiologic synchronization.^31–38^

## Methods

### Study design

**Figure 1:**
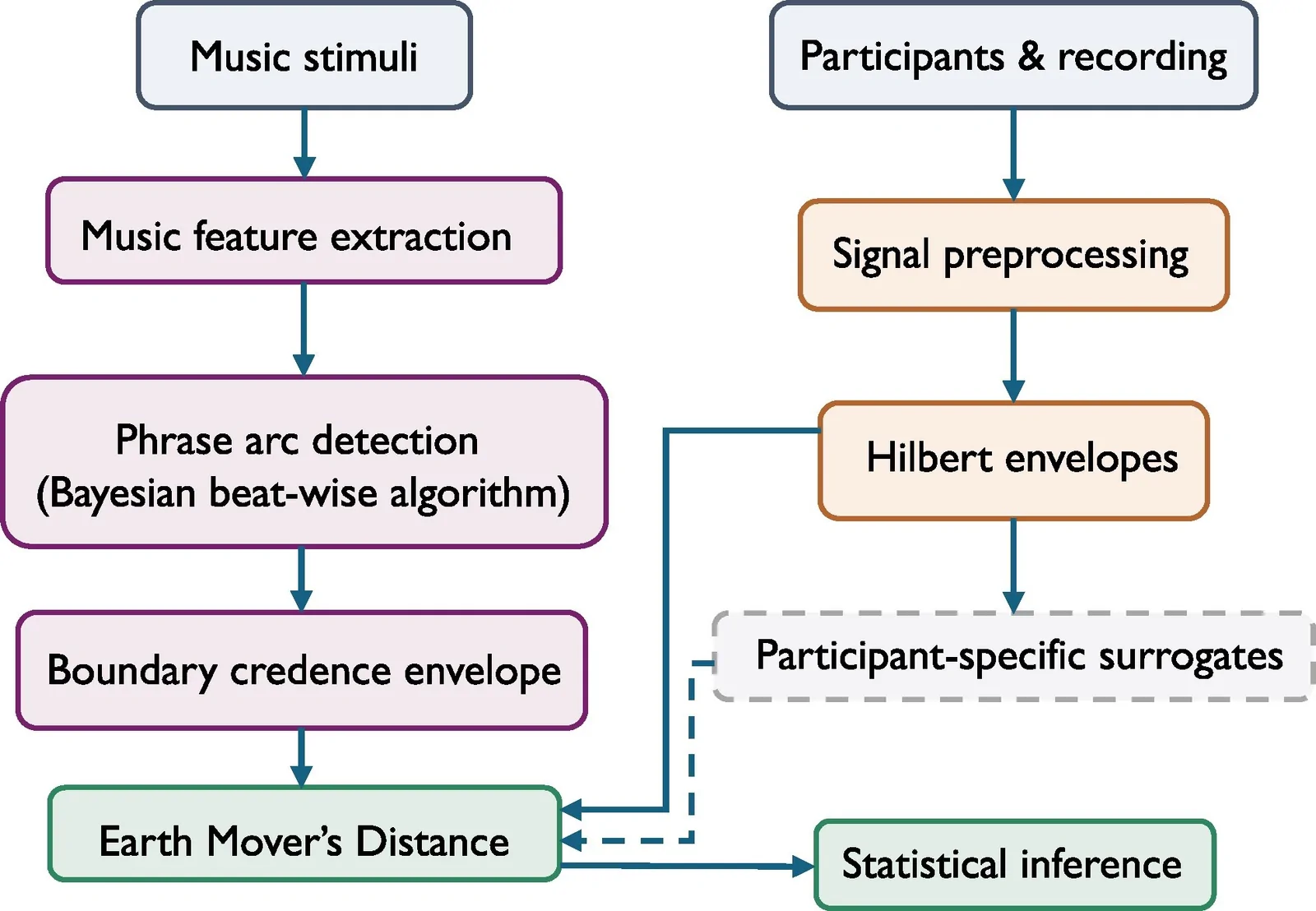
Analysis pipeline workflow showing the steps of how the music stimuli and the participant data were processed. Main computational steps shown along with arrows indicating which data was given as input for the subsequent steps. Detailed diagram in Supplementary Materials  *S2*.

Data were collected as part of the HeartFM study,^30^ which recruited 127 participants [75 women, mean age 43.3 (95% CI: 40.5–46.0)]. The workflow of the analysis is described in *Figure 1*. Participants were recruited as healthy volunteers or participants with hypertension under the HeartFM protocol. Eligibility required capacity to consent, ability to complete the listening protocol, and adequate hearing to perceive the musical stimuli. Participants taking beta-blockers were excluded because beta-adrenergic blockade may alter autonomic cardiovascular responses. Each participant listened to a selection of 9 tracks drawn from a larger set of 30 solo piano performances (8 original + 22 feature-modulated versions), rendered via a reproducing piano to ensure consistent expressive playback. Modulations altered tempo and/or loudness within pieces, enabling controlled investigation of feature-specific effects while preserving stylistic coherence. Across tracks, the averaged range was 27–199 beats per minute (tempo) and 7–23 sones (loudness). Participants completed demographic questions, a reduced Goldsmith’s Music Sophistication Index, and we also recorded any information they gave on whether or not they suffered from any medical conditions. Following a 5-min baseline, participants listened to a playlist of 9 tracks randomly selected from the 30. All tracks were different pieces, except the first and last tracks, which were different versions of the same piece. Respiration, ECG, and continuous BP were recorded throughout.

Across the repertoire, pieces differed in temporal structure, phrase organization, and dynamic contour. Accordingly, tracks varied in phrase regularity and in the density and magnitude of expressive changes, providing a wide range of predictability environments. This variability allowed us to test whether entrainment differs between tracks with smoother, more regular phrase arcs vs. those with more irregular or elongated expressive segments.

### Data acquisition

ECG was recorded using a Polar H10 sensor at 1000 Hz; RR intervals were extracted and down-sampled to 130 Hz by the Polar software. Respiration was measured using a BIOPAC thoracic belt at 13 Hz. Continuous BP was recorded using a CNAP 500 monitor (arm and finger cuffs).

ECG and respiration were aligned using the HeartFM^39^ recording software, whereas BP was synchronized retrospectively. Specifically, the BP recording was aligned to the common session endpoint and then segmented using the known track start times and track durations from the HeartFM listening protocol. More details on the alignment of BP to the other signals can be found in Supplementary Materials  *S3A*.

Music features were extracted from MIDI and audio using the Cosmodoit toolbox.^40^ Tempo and loudness were computed at the beat level, with manual correction of any misalignments to support beat-resolved analysis.

### Music boundary data

**Figure 2:**
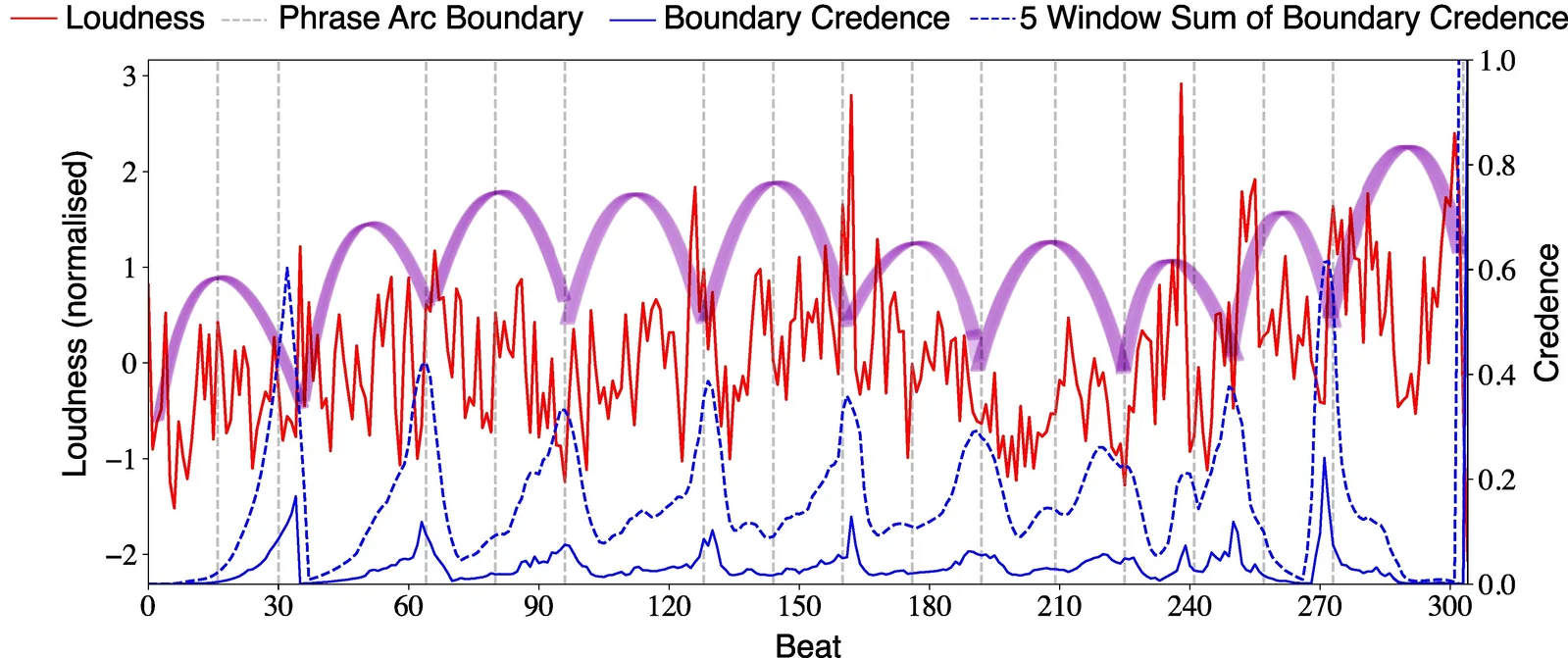
Example Bayesian arc-detection output for a track’s loudness and the resulting beat-wise boundary credence profile (the two bottom lines, where the dotted one shows the 5 window sum of the solid line). Phrase arcs inferred from boundary peaks are indicated for illustration as parabolic shapes with thicker lines.

Phrase arcs, which denote arc-like increases and decreases in music features, were identified using a Bayesian arc-detection algorithm.^29^ Expressive music features—such as loudness or tempo—are modelled as sequences of smoothly evolving phrase arc boundary placement probabilities (highest probabilities indicated by the peaks in the blue line in *Figure 2*). Within each phrase, these features tend to follow curved trajectories (e.g. gradual acceleration followed by deceleration, or crescendos followed by diminuendos), which are highlighted with purple arcs in *Figure 2*, and the model determines the most likely placements of the start and end-points of these curves (peaks in the blue line). The output from the algorithm is essentially a probability mapping of these curve boundaries based on the beat-time axis. Further details on the algorithm are provided in Supplementary Materials  *S3B*.

### Data preprocessing

Physiologic signals were pre-processed prior to analysis. Respiration baseline shifts were reduced using a rolling average, and a fourth-order Butterworth low-pass filter (1 Hz cut-off) was applied to remove high-frequency noise. RR intervals were manually inspected and corrected where needed. RR interval artefacts were identified using a centred rolling-median rule: intervals deviating by more than 30% from an 11-beat rolling median were treated as artefacts. BP waveform signals were used without additional preprocessing beyond segmentation. A total of 127 participants completed data collection. One participant was removed after data collection due to medication they were taking, leaving 126 participants in the retained analysis cohort. Participants with known arrhythmia or unreliable RR interval series were excluded from RR-based analyses. Participants taking beta-blockers were excluded. Other medication use was recorded where available, but medication data were not complete enough for formal covariate modelling. Because physiological recordings were acquired from separate devices, signal availability differed by channel. Usable continuous BP data were available for 116/126 participants, ECG-derived RR interval data for 125/126 participants, and respiration data for 110/126 participants. Missing or unusable data in one physiological channel did not lead to complete participant exclusion, provided that the participant had usable data for the remaining physiological signals. Therefore, analyses were conducted using signal-specific available-case samples, with the denominator for each analysis reflecting the number of participants with usable data for that physiological channel.

Because the music boundary output is beat-indexed, all signals (music and physiologic) were interpolated to the shared beat-time grid. Signals were normalized using median and inter-quartile range to reduce scale differences while preserving relative temporal structure.^41^ To represent physiologic dynamics in a form comparable to the music boundary envelope, envelopes of respiration, RR intervals, and BP waveform were computed using the Hilbert transform. This provided smooth, continuous amplitude envelopes suitable for comparison with the boundary credence profiles.

### Entrainment calculation

Synchrony between physiologic responses and music structures was quantified using Earth Mover’s Distance (EMD), implemented in Python. EMD measures the minimal cost required to transform one distribution into another; in this context, it captures similarity between the shape of a music boundary credence profile and a physiologic envelope. Lower EMD indicates stronger alignment (greater entrainment). Further details on alternative metrics tested and the rationale for selecting EMD as the primary morphology-based alignment metric are provided in Supplementary Materials  *S3C*.

### Statistical analysis

To assess whether observed alignment exceeded naturally occurring fluctuations in physiology, a participant-specific surrogate null model was implemented.^42,43^ For each participant and target track, physiological data from the participant's other tracks, excluding baseline and the track under analysis, were concatenated. Surrogate segments matched in duration to the target track were randomly sampled from the concatenated signal. This preserved participant-specific physiological characteristics while breaking the temporal link between the physiological signal and the specific music stimulus. EMD was computed between the music boundary envelope and each surrogate physiological segment over 1000 iterations per participant and track.

Kolmogorov–Smirnov tests were used as an initial descriptive analysis of distributional separation between original and surrogate EMD values. To specifically test whether the original EMD was lower than the surrogate EMD, the primary surrogate analysis used a participant-specific percentile statistic. Further details are provided in Supplementary Materials  *S3D*. Relationships between entrainment strength and phrase-arc summary statistics, including mean, standard deviation, and maximum arc length, were examined using the Pearson correlation. Similarity between loudness and tempo arc structures was also assessed. Multiple-comparison correction was applied within the relevant families of tests using Bonferroni correction and/or the Benjamini–Hochberg false discovery rate (FDR), as appropriate for each analysis.

As each participant contributed repeated observations across tracks and music–physiology pairings, mixed-effects models were fitted as sensitivity analyses. The models used the six EMD pairings: loudness–respiration, loudness–RR intervals, loudness–BP waveform, tempo–respiration, tempo–RR intervals, and tempo–BP waveform. Higher EMD values indicate weaker alignment, whereas lower EMD values indicate stronger alignment. Full model specifications are provided in Supplementary Materials  *S3E*.

## Results

### Participant-specific surrogate analysis shows heterogeneous evidence for stimulus-locked alignment

When examined by signal pairing, significant original-vs.-surrogate distribution difference was most prevalent for tempo pairings: 25 of 30 tracks for tempo–RR (T–RR), 21 for tempo–BP waveform (T–BPW), and 16 for tempo–respiration (T–R). Loudness pairings also showed significant separation in 18 of 30 tracks for loudness–RR (L–RR), 16 for loudness–BP waveform (L–BPW), and 8 for loudness–respiration (L–R). Detailed track-by-track Kolmogorov-Smirnov results are provided in Supplementary Materials  *S4*.

The participant-specific percentile analysis provided a conservative test of whether original EMD values were lower than expected from each participant's own surrogate physiology. Across all tracks and pairings, the pooled effect was centred close to the surrogate null rather than showing a uniform original-lower-than-surrogate pattern. The overall mean percentile was (*u* = 0.510), with Cliff's (*δ*=−0.020). Using the wider track-clustered interval, the 95% CI for (*u*) was 0.495 to 0.526, corresponding to a Cliff's *δ* interval of −0.052 to 0.011. Thus, the primary pooled surrogate analysis did not support a global stimulus-locking effect across the full dataset.

Importantly, this pooled result masked substantial heterogeneity across both music–physiology pairings and individual tracks. The strongest pooled effect in the expected direction was observed for tempo–BP waveform pairings (*u* = 0.471, Cliff's *δ* = 0.058), although this effect weakened when uncertainty was clustered by track. Within this pairing, some individual track versions showed much larger effects, indicating that original-lower-than-surrogate alignment was concentrated in specific musical stimuli rather than uniformly distributed across the repertoire. Respiration and RR interval pairings showed weaker or opposite original-vs.-surrogate patterns. These findings suggest that entrainment is not a uniform response to music, but may be influenced by feature, signal, and track; with BP waveform responses showing the clearest trend of original-lower-than-surrogate alignment.

**Figure 3:**
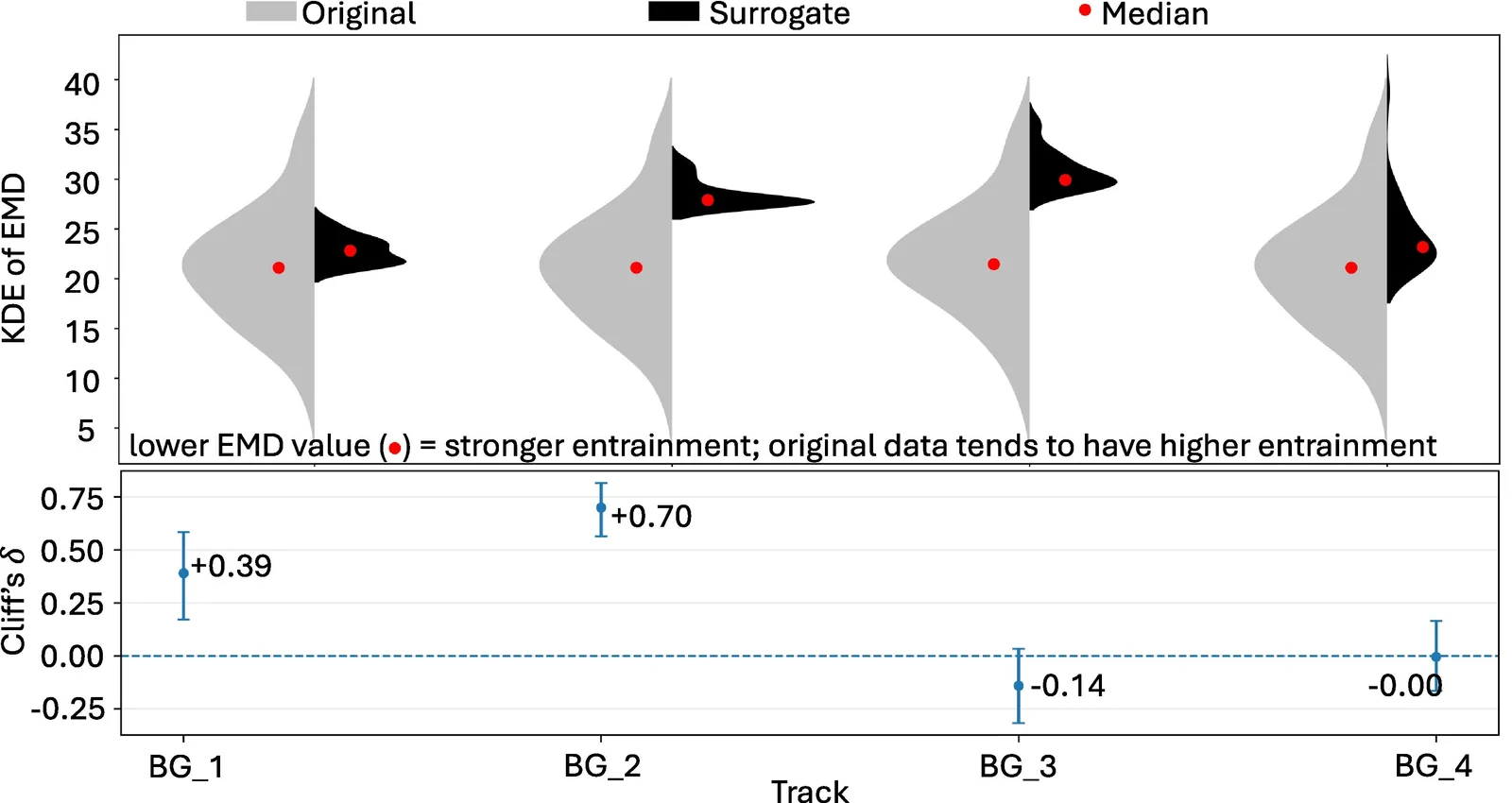
Entrainment results (EMD) between music boundary credence envelopes and physiologic envelopes for tempo-BP waveform for all versions of one piece. Top shows a violin plot for each of the four versions for a selected track. Original data (left side) and participant-specific surrogate distributions (black), with median values marked, showing lower distance between music-physioogy for the original data. Lower EMD indicates stronger alignment. Bottom plot shows corresponding Cliff′s δ for the effect sizes of the results shown in the violin plots. Positive Cliff's δ indicates lower original EMD than participant-specific surrogate EMD. Larger effects were observed for selected track versions, particularly BG_1 and BG_2, illustrating the track-specific nature of the surrogate effect.

For example, *Figure 3* illustrates the tempo–BP waveform values for the Bach-Gounod's Ave Maria, which showed larger track-level effects than the tempo–BP waveform estimate pooled for the entire dataset, highlighting that the surrogate effect was driven by selected track versions rather than by a uniform response across all stimuli. Therefore, the surrogate analysis was interpreted as an initial hypothesis test rather than as the sole criterion for physiological relevance. The absence of a robust pooled surrogate effect does not preclude systematic differences in EMD-based entrainment between musical features, versions, or phrase structures, which are examined below.

### Versions of a piece can differ in entrainment strength

**Figure 4:**
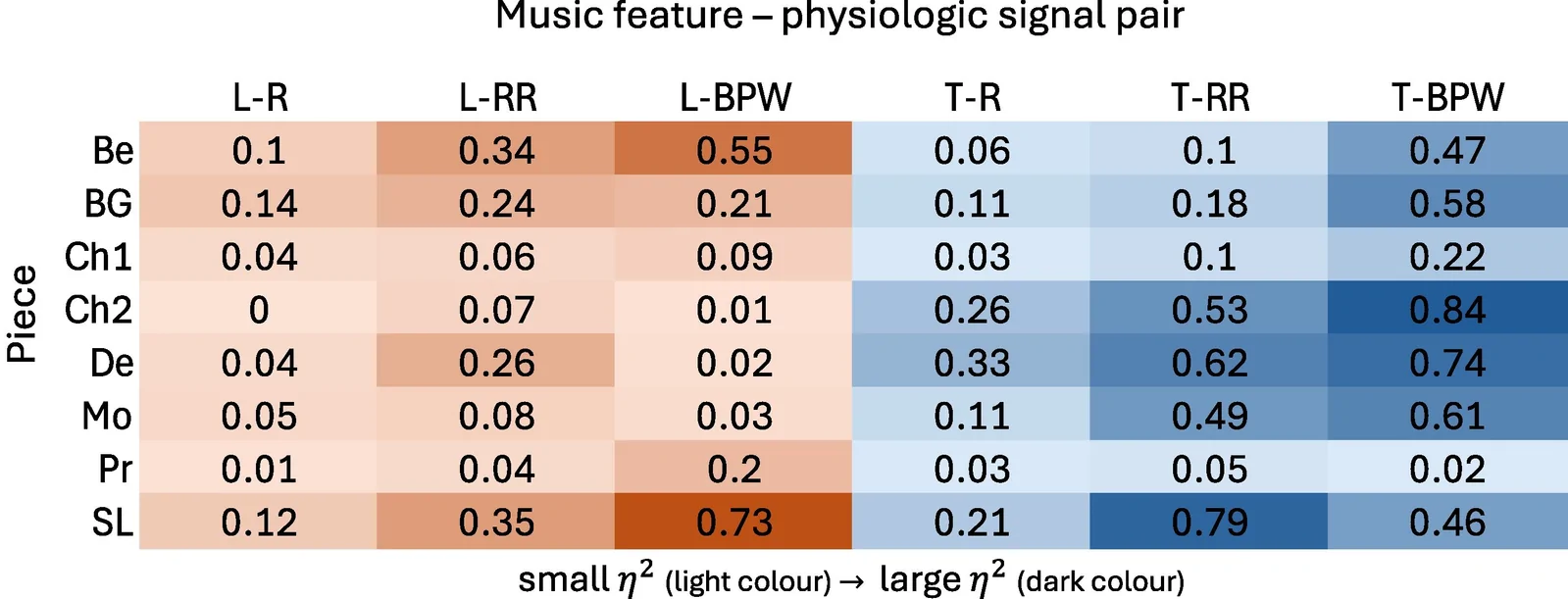
ANOVA results for entrainment outcomes across versions within each piece. Large effect sizes are indicated by darker colours. Stronger between-version differences are observed for several tempo–physiology pairings. BPW: BP waveform; L: loudness; T: tempo; R: respiration; RR: RR intervals. Detailed results table in Supplementary Materials  *S5*.

Analysis of variance (ANOVA) comparing original EMD values across versions within each piece revealed version effects for several pieces, particularly for tempo-physiology pairings (*Figure 4*). Effect sizes ranged from negligible ($\eta^{2}$ < 0.05) to large ($\eta^{2}$ > 0.50). The strongest effects were observed for tempo–BP pairings, highlighting its influence on this cardiovascular signal.

For pieces showing version effects, original or minimally modified versions (versions 1–2) generally produced lower mean EMD values than more heavily modified versions (versions 3–4), although this pattern was not universal.

### Loudness is a stronger entrainment driver than tempo

Comparing loudness–physiology and tempo–physiology EMD distributions across tracks revealed lower EMD values (stronger alignment) for loudness. Because EMD differences were paired within participants and exhibited non-normal distributions, statistical comparisons were performed using the Wilcoxon signed-rank test rather than parametric t-tests. Using paired Wilcoxon signed-rank tests with FDR correction (q < 0.05), loudness–physiology EMD values were significantly lower than tempo–physiology values in 29 of 30 tracks when looking at the RR interval and BP response, and for respiration in 27 of 30 tracks. Detailed results are provided in Supplementary Materials  *S6*.

### Predictability of phrase structure is correlated with entrainment

To assess whether phrase-level organization relates to entrainment strength, we examined summary statistics of phrase arcs derived from the Bayesian boundary envelopes. Predictability was operationalized through (i) mean arc length and (ii) variability of arc length, reflecting how regularly phrase transitions recur across a track. Pearson correlations between EMD values and phrase arc summary statistics revealed significant associations for both loudness–physiology and tempo–physiology pairings (*Table 1*). These positive correlations show that increasing arc length and variability are associated with higher EMD (weaker alignment), indicating that more compact and regular phrase organization supports stronger entrainment.

**Table 1:** Pearson correlations between music–physiology EMD and phrase-arc summary statistics

Grouping		Statistical test	
EMD Pair	Phrase Arc Feature	Pearson’s r	*P*-value
L-P	L-T similarity	0.59	8.96E-04
T-P	L-T similarity	0.93	8.49E-14
L-P	Max arc length	0.87	3.05E-08
T-P	Max arc length	0.91	7.99E-12
L-P	Mean arc length	0.84	1.28E-07
T-P	Mean arc length	0.67	5.54E-05
L-P	Std arc length	0.82	3.92E-07
T-P	Std arc length	0.75	2.66E-06

Correlations observed between predictability metrics (arc features and entrainment). L: loudness; T: tempo; P: physiology.

Similarity between loudness and tempo arc structures (quantified by the EMD between the two boundary credence envelopes) was also correlated with entrainment strength, showing the importance of simultaneous changes in music features for improved synchrony. This influence was stronger for tempo–physiology pairings (*r* = 0.93, *P* = 8.49E-14) compared to loudness–physiology pairings (*r* = 0.59, *P* = 8.96E-04), indicating that loudness affects entrainment with tempo more than vice versa.

### Influence of covariates and multiple testing

#### Participant-level covariates

To address whether confounding variables included in the demographic data influenced entrainment strength, we performed an exploratory analysis linking these data to real EMD values for each participant-track observation. We examined familiarity, music sophistication, body mass index (BMI), age, and gender as participant-level covariates. Because participants contributed repeated observations across tracks, Spearman correlations and group comparisons were considered exploratory. Of all the variables, only familiarity had effects that survived FDR. The same trend was seen in the mixed-effects models. Relative to unfamiliar tracks, highly familiar tracks showed higher EMD values (lower entrainment) for tempo pairings. Omnibus Kruskal-Wallis and ANOVA tests similarly indicated significant familiarity effects for tempo pairings as well. Mixed-effects models showed no significant effects for music sophistication, BMI, age, or gender on any EMD metric after correction for multiple comparisons.

#### Mixed-effects sensitivity analyses

To account for repeated observations within participants and track stimuli, we fitted mixed-effects models with participant and track stimulus as random intercepts. In the original-only model, tempo-based pairings showed significantly higher EMD than loudness-based pairings (*β* = 11.77, FDR-corrected *P* < 0.001). Because higher EMD indicates weaker alignment, this supports the finding that loudness phrase structure produced stronger entrainment than tempo phrase structure after accounting for repeated measures.

Track position during listening sessions was not significantly associated with EMD (*β* = 0.012, *P* = 0.978), providing no evidence for a systematic fatigue or session-order effect.

We also fitted an original-minus-surrogate delta model, where negative values indicate lower EMD for the original physiological signal than for the participant-specific surrogate. This model showed that differences between original and surrogate were not uniform across physiological channels. The clearest lower delta values were observed for loudness-BP and tempo-BP pairings, while respiration and RR interval pairings showed more mixed effects.

## Discussion

### Study and analysis design

#### Stimulus choice and generalizability

The use of Western classical solo piano is an important limitation, and the present results should not be assumed to generalize automatically to all musical genres or cultural contexts. However, the focus of the analysis is not Western classical style *per se*, but the expressive prosodic features through which music communicates affect: changes in loudness, tempo, timing, and phrase shape. These features are closely related to acoustic cues used in speech prosody and affective communication, where intensity, rate, timing, and pitch-related changes contribute to emotional meaning. Therefore, the present study should be interpreted as testing whether acute autonomic dynamics can be mapped to expressive phrase structures under controlled stimulus conditions. Future work may extend this framework to other repertoires and participant groups to determine which aspects of music-physiology entrainment are general and which are style- or culture-specific.

#### Interpretation of EMD-based alignment

The sensitivity analyses clarify what the EMD metric is, and is not, measuring. The weaker findings from cross-correlation and lagged cross-correlation suggest that the observed effect is not simply a fixed-lag linear correlation between music features and physiology,^6,36^ but an effect changing accordingly to the music stimulus. Similarly, the weaker coherence findings indicate that the solo-piano stimuli may not produce the same sustained frequency-specific coupling observed previously for vocal opera excerpts.^44^ This is plausible because the earlier Verdi stimuli contained prominent vocal and phrase structures close to cardiorespiratory timescales, whereas the present solo-piano excerpts vary more in phrase timing and expressive contour. Thus, EMD may be most appropriate here as a morphology-based measure of alignment between musical boundary structure and acute physiological response envelopes.

### Surrogate testing indicates feature and signal-specific alignment

The participant-specific surrogate analysis provided a conservative test of whether physiology–music alignment exceeded participant-specific physiological fluctuations. This analysis did not show robust global evidence that original EMD values were lower than surrogate EMD values across all tracks and pairings. Instead, original-vs.-surrogate effects were heterogeneous across physiological channels, with the strongest trend observed for tempo–BP waveform pairings. This result clarifies the interpretation of the feature-level findings. Loudness, tempo, version manipulation, and phrase predictability were associated with systematic differences in morphology-based music–physiology alignment. The participant-specific surrogate model is stringent: it preserves each individual's physiologic characteristics and typical response patterns during the same recording session while breaking the temporal link to the specific track being analysed. Stronger alignment in original vs. surrogate conditions could therefore indicate that entrainment depends on the precise timing and organization of phrase boundaries as they unfold. Physiologic envelopes track boundary signals more closely than the surrogate envelopes, suggesting that autonomic dynamics are organized around perceived structural moments. This is consistent with theories proposing that phrase-level organization guides attention, expectation, and embodied regulation.^22–24,45^ This strengthens the observation that entrainment reflects sensitivity to musically meaningful structure rather than a by-product of physiologic fluctuations.

The variation in original-vs.-surrogate separation across tracks and versions further reinforces that entrainment is not a uniform ‘music effect’ but is sensitive to the specific structural properties of a performance or recording. This heterogeneity suggests that entrainment depends on how phrase cues are distributed, how strongly they are marked, and potentially how they interact with listener expectations learned from experiences of music styles and structures.

### Expressive manipulations change entrainment strength by altering phrase coherence

Seeing that different versions of a piece produce different entrainment strengths reveals that autonomic alignment is sensitive to how expressive features are organized, not merely to which piece is being played. Versions preserve music content while modifying tempo and/or loudness; between-version differences are most plausibly driven by changes in phrase-level coherence and predictability (how clearly and consistently expressive features mark structural boundaries).

This interpretation aligns with predictive engagement theories: listeners continuously form expectations about when structural events will occur, and phrase-level coherence stabilizes these expectations.^31–34^ When expressive manipulations disrupt the internal relationship between dynamics and timing, or shift the placement and strength of boundaries, they may reduce the clarity of structural cues and weaken physiologic alignment.

The finding that original and minimally modified versions often outperformed more heavily modified versions suggests that there may be limits to how much expressive features can be altered before phrase coherence degrades. This has practical implications: future music intervention design should treat tempo or loudness as independent parameters to consider their role in maintaining phrase-level intelligibility and expressive authenticity. Some pieces showed stronger version effects than others, potentially reflecting differences in baseline phrase regularity or in how central specific expressive cues are to each piece's structural identity.

Additionally, perceptual factors such as reduced authenticity or an ‘uncanny’ performance quality may contribute if the modulations create mismatches between expected and heard expressive contours.^46^

### Why loudness is a stronger entrainment driver than tempo

The consistent advantage of loudness over tempo across 27 of 30 tracks reflects fundamental differences in how these features are perceived and processed. Loudness provides immediate salience cues: intensity changes are perceived with short latency (50–100 ms)^47^ and can mark structural boundaries with high temporal precision. Abrupt dynamic shifts or local peaks in loudness contour create prominent perceptual events that can anchor autonomic responses.

Tempo, in contrast, is typically derived from the tactus, a felt sense of pulse that emerges over multiple beats.^48–50^ Detecting tempo changes requires integrating timing information across longer windows, making tempo a less temporally precise cue for moment-to-moment physiologic alignment. While tempo boundaries can support entrainment, they do so at a coarser temporal grain, which may explain the weaker overall entrainment observed.

The strong correlation between tempo entrainment and loudness-tempo structural similarity (*r* = 0.93) reveals an additional mechanism: tempo-related entrainment is amplified when tempo phrasing is reinforced by congruent loudness phrasing. When both features mark boundaries at the same moments, listeners receive multiple congruent cues that a phrase transition is occurring. This cross-feature congruence likely increases perceptual certainty about structural segmentation and thereby strengthens autonomic alignment.

The exceptions to the loudness-dominance pattern: De_1 (Debussy playing Debussy's Sunken Cathedral) for respiration; and Ch1_3 (Reisenaur playing Chopin's Berceuse) and Mo_3 (Landowska playing Mozart) for all three physiological signals warrant consideration. Examining outliers in future work could clarify boundary conditions for feature-specific entrainment.

### No single autonomic channel dominates entrainment

The finding that BP response to the loudness and tempo phrase structures showed strong and consistent response suggests that the relative strength of alignment varies with track structure, feature organization, and potentially with the timescale at which each signal can adapt.

Respiration operates on a slower cycle (∼3–5 s per breath) compared to cardiac intervals (∼1 s per beat), which may constrain how closely respiration can entrain to beat-level phrase boundaries. Cardiovascular signals, with faster underlying cycles, may be better positioned to adapt continuously to unfolding music structures. Additionally, respiratory sinus arrhythmia—the modulation of heart rate by breathing—means that respiration-related dynamics propagate into RR intervals and BP.^51^ Future work using time-frequency coherence could help disentangle cardio-respiratory effects and clarify whether different signals align at different timescales or through different mechanisms.

### Predictability enhances entrainment through the anticipation of phrase transitions

The correlations between phrase arc regularity (mean arc length, arc length variability) and entrainment strength support the interpretation that temporal anticipation enhances music-physiology coupling. Tracks with shorter, more consistent phrase arcs provide repeated structural cues within a single listening span, enabling listeners to build and refine internal models of when boundaries will occur.^35–38^ When structural timing becomes predictable, autonomic dynamics may align more tightly because the body can ‘prepare’ for upcoming transitions.

Conversely, tracks with long or highly variable arcs offer fewer stable reference points for prediction. Without sufficient regularity to support anticipation, listeners may experience phrase boundaries as more surprising or ambiguous events, which could reduce the strength and consistency of physiologic alignment. The finding that maximum arc length also correlates with entrainment (longer max arcs associate with weaker entrainment) is consistent with this interpretation: tracks dominated by a few very long arcs have fewer boundary events overall, reducing opportunities for learning and prediction. While physiology might respond to these phrase boundaries, it cannot entrain as well.

This predictability framework also helps explain version effects. Music modifications introduced to create the different versions changed the priors for the phrase arcs, which altered their predictability.

An important methodological caveat is that we indexed predictability post-hoc using the phrase arc statistics themselves rather than from independent listener judgements or computational models of expectation. While the correlations are robust, they reflect associations between structural regularity and entrainment rather than direct evidence that prediction mechanisms mediate the relationship. Future work incorporating listener expectancy ratings or computational models of music expectation could provide stronger causal evidence for the role of anticipation in entrainment.

### Covariate effect analyses support the main conclusions

Analyses testing demographic variables and musical sophistication showed no significant effects on the EMD quantified entrainment. This is in line with previous work showing that musical training does not significantly change the way the autonomic system entrains to the music.^44^ Even though familiarity was associated with weaker tempo-based alignment, this pattern does not explain the main feature-level findings, including the loudness-vs.-tempo difference. These results are consistent with morphology-based alignment differences being driven primarily by musical structure rather than broad participant-level covariates. This may suggest that familiar music evokes less direct physiological tracking of tempo, perhaps because listeners rely more on memory, prediction, or internally generated expectations rather than moment-to-moment acoustic-temporal alignment.^52,53^ The absence of significant MSI, BMI, age, and gender effects suggests that these entrainment differences were more strongly related to stimulus familiarity than to broad demographic or individual-difference characteristics. These results support the interpretation that observed alignment is driven primarily by temporal organization of the music stimulus rather than by confounding participant-specific variables.

Together, the mixed-effects analyses support three main conclusions: the loudness-vs.-tempo effect remained robust after accounting for repeated measures; there was no evidence that entrainment changed systematically across the listening session; and familiarity did not explain stronger alignment.

### Potential implications and future directions

The present findings characterize music–physiology coupling as a measurable, structure-dependent phenomenon; they have yet to establish clinical benefit. Autonomic alignment varied systematically with expressive features, which suggests that music might be treated as a feature-specific stimulus for engaging autonomic activity rather than as a generic relaxation aid.

The participant-specific surrogate analysis revealed substantial between-person heterogeneity in entrainment strength. In principle, such variation could help distinguish individuals by sensitivity to the music stimulus, although we did not test whether it relates to any clinical outcome. Considered alongside established measures such as heart rate variability (HRV), music-evoked entrainment may offer complementary information: whereas resting HRV reflects baseline autonomic tone, stimulus-evoked entrainment reflects dynamic responsiveness to a controlled input. This interpretation is hypothetical and would require dedicated validation before entrainment could be regarded as a cardiovascular adaptability index.

A central open question is whether the acute alignment observed here bears any relationship to longer-term autonomic function. This cannot be addressed with the present cross-sectional, single-session data. Future longitudinal work could examine whether individuals with stronger acute responses show also stronger changes following repeated structured listening, and whether feature parameters (e.g. phrase length, loudness contour, predictability) can be adjusted to influence coupling. Were such effects demonstrated, the computational pipeline described here could help design individualized listening protocols for study in that setting. Any therapeutic application would need to be established through prospective trials with defined cardiovascular endpoints.

### Limitations

Several limitations should be noted. The cohort was not a defined cardiovascular disease intervention cohort, no clinical endpoints were assessed, and the study used a single-session listening design. Generalizability beyond Western classical solo piano remains uncertain, though the study looked at prosody which cuts across cultures and genres. BP timing alignment was retrospective rather than hardware-triggered, and medication data beyond beta-blocker exclusion were incomplete. Finally, predictability was inferred from phrase-arc statistics rather than independent listener expectancy ratings. These limitations mean that the findings should be interpreted as evidence of the acute music–physiology relationship facilitated through the mechanism of entrainment rather than evidence of therapeutic cardiovascular benefit.

## Conclusion

This study shows that expressive musical phrase structure can be mapped to acute autonomic dynamics during naturalistic listening. Loudness-based phrase structure showed stronger morphology-based entrainment than tempo-based phrase structure across most tracks, and phrase predictability and cross-feature congruence were associated with stronger alignment. Participant-specific surrogate testing indicated that original-vs.-surrogate effects were heterogeneous rather than uniformly present across physiological channels, with the strongest trend observed for tempo–BP waveform pairings. These findings support a computational framework for studying how structured musical features relate to cardiorespiratory dynamics, while also showing that evidence for stimulus-locking depends on the physiological channel. Together these findings connect expressive music organization to measurable autonomic dynamics using phrase arcs, EMD-based similarity, and within-participant surrogates. They characterize music–physiology coupling as a structure-dependent, quantifiable phenomenon under controlled listening conditions; they do not, on their own, establish clinical benefit. Whether targeting boundary strength, predictable structural organization, and music-feature coherence can improve music-based interventions is a hypothesis for future work. The computational pipeline described here provides a basis for extending these analyses to other genres, populations, and, ultimately, clinical settings, where prospective studies with defined cardiovascular endpoints would be needed to test any therapeutic value.

## Supplementary Material

qyag149_Supplementary_Data

## Data Availability

The datasets generated and/or analysed during the current study are not publicly available as they are part of a larger planned data publication. Until public release, data are available from the corresponding author upon reasonable request. No third-party datasets were analysed.

## Lead author biography

**Natalia Cotic** is a PhD student in Biomedical Engineering whose research examines physiological and musical signal interactions within the HeartFM project. Her work focuses on computational modelling of heart rate variability, respiration, and blood pressure in response to performed and composed musical structures. She holds a Bachelor's and Master's degree in Biomedical Engineering from King's College London, where her research centred on real-time convolutional neural network-based denoising of catheterisation laboratory images.

## References

[1] WHO . Cardiovascular diseases [Internet]. 2025. Available from: https://www.who.int/news-room/fact-sheets/detail/cardiovascular-diseases-(cvds) (10 March 2026, date last accessed).

[2] Kulinski J, Ofori EK, Visotcky A, Smith A, Sparapani R, Fleg JL. Effects of music on the cardiovascular system. Trends Cardiovasc Med 2022;32:390–8. 34237410 10.1016/j.tcm.2021.06.004 PMC8727633

[3] Mandel SE, Hanser SB, Secic M, Davis BA. Effects of music therapy on health-related outcomes in cardiac rehabilitation: a randomized controlled trial. J Music Ther 2007;44:176–97. 17645384 10.1093/jmt/44.3.176

[4] Koelsch S, Jäncke L. Music and the heart. Eur Heart J 2015;36:3043–9. 26354957 10.1093/eurheartj/ehv430

[5] Teckenberg-Jansson P, Turunen S, Polkki T, Lauri-Haikala MJ, Lipsanen J, Henelius A et al Effects of live music therapy on heart rate variability and self-reported stress and anxiety among hospitalized pregnant women: a randomized controlled trial. Nord J Music Ther 2019;28:7–26.

[6] Nomura R . Reliability for music-induced heart rate synchronization. Sci Rep 2024;14:12200. 38806616 10.1038/s41598-024-62994-0 PMC11133398

[7] Loomba RS, Arora R, Shah PH, Chandrasekar S, Molnar J. Effects of music on systolic blood pressure, diastolic blood pressure, and heart rate: a meta-analysis. Indian Heart J 2012;64:309–13. 22664817 10.1016/S0019-4832(12)60094-7 PMC3860955

[8] Mir IA, Chowdhury M, Islam RM, Ling GY, Chowdhury AABM, Hasan ZM et al Relaxing music reduces blood pressure and heart rate among pre-hypertensive young adults: a randomized control trial. J Clin Hypertens 2020;23:317–22. 10.1111/jch.14126 PMC8029898 33347732

[9] Chuen L, Sears D, McAdams S. Psychophysiological responses to auditory change. Psychophysiology 2016;53:891–904. 26927928 10.1111/psyp.12633

[10] Burns JL, Labbe E, Arke B, Capeless K, Cooksey B, Steadman A et al The effects of different types of music on perceived and physiological measures of stress. J Music Ther 2002;39:101–16. 12213081 10.1093/jmt/39.2.101

[11] Parada-Cabaleiro E, Batliner A, Schuller BW. The effect of music in anxiety reduction: a psychological and physiological assessment. Psychol Music 2020;49:1637–53.

[12] Mockel M, Rocker L, Stork T, Vollert J, Danne O, Eichstadt H et al Immediate physiological responses of healthy volunteers to different types of music: cardiovascular, hormonal and mental changes. Eur J Appl Physiol Occup Physiol 1994;68:451–9. 7826431 10.1007/BF00599512

[13] Knight WEJ, Rickard NS. Relaxing music prevents stress-induced increases in subjective anxiety, systolic blood pressure, and heart rate in healthy males and females. J Music Ther 2001;38:254–72. 11796077 10.1093/jmt/38.4.254

[14] Hu X, Li F, Ng J. On the relationships between music-induced emotion and physiological signals. In: The 19th International Society for Music Information Retrieval Conference (ISMIR 2018; Paris, France). 2018.

[15] Cross I . Music and communication in music psychology. Psychol Music 2014;42:809–19.

[16] Bernardi L, Porta C, Casucci G, Balsamo R, Bernardi NF, Fogari R et al Dynamic interactions between musical, cardiovascular, and cerebral rhythms in humans. Circulation 2009;119:3171–80. 19569263 10.1161/circulationaha.108.806174

[17] Bernardi NF, Codrons E, Di Leo R, Vandoni M, Cavallaro F, Vita G et al Increase in synchronization of autonomic rhythms between individuals when listening to music. Front Physiol 2017;8:785. 29089898 10.3389/fphys.2017.00785 PMC5651050

[18] Kosta K, Ramirez R, Bandtlow OF, Chew E. Mapping between dynamic markings and performed loudness: a machine learning approach. J Math Music 2016;10:149–72.

[19] Kosta K, Bandtlow OF, Chew E. Dynamics and relativity: practical implications of dynamic markings in the score. J New Music Res 2018;47:438–61.

[20] Rink J . Playing in time: rhythm, metre and tempo in Brahms’s Fantasien op. 116. In: The Practice of Performance: Studies in Musical Interpretation. Cambridge: Cambridge University Press; 1995. p254–82.

[21] Langner J, Goebl W. Visualizing expressive performance in tempo-loudness space. Comput Music J 2003;27:69–83.

[22] Todd NPM . The dynamics of dynamics: a model of musical expression. J Acoust Soc Am 1992;91:3540–50.

[23] Todd NPM . Motion in music: a neurobiological perspective. Music Percept 1999;17:115–26.

[24] Gabrielsson A, Juslin PN. Emotional expression in music performance: between the performer’s intention and the listener’s experience. Psychol Music 1996;24:68–91.

[25] Stowell D, Chew E.. Maximum a posteriori estimation of piecewise arcs in tempo time-series; From Sounds to Music and Emotions. CMMR 2012. Lecture Notes in Computer Science, vol 7900. Springer, Berlin, Heidelberg. 2013.

[26] Povel DJ . Temporal structure of performed music. Acta Psychol (Amst) 1977;41:309–20.

[27] Chuan CH, Chew E. A hybrid system for automatic generation of style-specific accompaniment. International Joint Workshop on Computational Creativity; London. 2007.

[28] Cheng E, Chew E. A local Maximum phrase detection method for analyzing phrasing strategies in expressive performances. Commun Comput Inf Sci 2009;37:347–53.

[29] Guichaoua C, Lascabettes P, Chew E. End-to-end Bayesian segmentation and similarity assessment of performed music tempo and dynamics without score information. Music Sci (Lond) 2024;7:20592043241233411.

[30] ERC . Maximizing the Therapeutic Potential of Music through Tailored Therapy with Physiological Feedback in Cardiovascular Disease [Internet]. 2020. Available from: 10.3030/957532 (10 March 2026, date last accessed).

[31] Levitin DJ . Why music moves us. Nature 2010;464:834–5.

[32] Levitin DJ, Chordia P, Menon V. Musical rhythm spectra from Bach to Joplin obey a 1/f power law. Proc Natl Acad Sci U S A 2012;109:3716–20. 22355125 10.1073/pnas.1113828109 PMC3309746

[33] Arnal LH, Giraud AL. Cortical oscillations and sensory predictions. Trends Cogn Sci 2012;16:390–8. 22682813 10.1016/j.tics.2012.05.003

[34] Cheung VKM, Harrison PMC, Meyer L, Pearce MT, Haynes JD, Koelsch S. Uncertainty and surprise jointly predict musical pleasure and amygdala, hippocampus, and auditory cortex activity. Curr Biol 2019;29:4084–92.e4. 31708393 10.1016/j.cub.2019.09.067

[35] Solli S, Danielsen A, Leske S, Blenkmann AO, Doelling KB, Solbakk AK et al Rhythm-based temporal expectations: unique contributions of predictability and periodicity. J Cogn Neurosci 2024;37:1–27. 10.1162/jocn_a_02261 39432692

[36] Eerola T . The dynamics of musical expectancy: cross-cultural and statistical approaches to melodic expectations [Internet]. 2003. Available from: https://urn.fi/URN:ISBN:951-39-1655-3 (10 March 2026, date last accessed).

[37] Stupacher J, Matthews TE, Pando-Naude V, Elst OFV, Vuust P. The sweet spot between predictability and surprise: musical groove in brain, body, and social interactions. Front Psychol 2022;13:906190. 36017431 10.3389/fpsyg.2022.906190 PMC9396343

[38] Singer N, Jacoby N, Hendler T, Granot R. Feeling the beat: temporal predictability is associated with ongoing changes in music-induced pleasantness. J Cogn 2023;6:34. 37457107 10.5334/joc.286 PMC10348017

[39] Chew E, Fyfe L, Picasso C, Lambiase P. Seeing music’s effect on the heart. Eur Heart J 2024;45:4359–63. 39405174 10.1093/eurheartj/ehae436

[40] Guichaoua C, Bedoya D, Chew E. Cosmodoit: A Python Package for Adaptive, Efficient Pipelining of Features from Performed Music [pre-print, 2026].

[41] De Amorim LBV, Cavalcanti GDC, Cruz RMO. The choice of scaling technique matters for classification performance. Appl Soft Comput 2022;133:109924.

[42] Lancaster G, Iatsenko D, Pidde A, Ticcinelli V, Stefanovska A. Surrogate data for hypothesis testing of physical systems. Phys Rep 2018;748:1–60.

[43] Theiler J, Eubank S, Longtin A, Galdrikian B, Farmer JD. Testing for nonlinearity in time series: the method of surrogate data. Physica D 1992;58:77–94.

[44] Cotic N, Pope V, Lambiase PD, Chew E. Autonomic entrainment to music structure in Verdi opera. Eur Heart J Imaging Methods Pract 2026;4:qyag025. 41808739 10.1093/ehjimp/qyag025 PMC12968618

[45] Lerch A, Arthur C, Pati A, Gururani S. An interdisciplinary review of music performance analysis. Trans Int Soc Music Inf Retr 2020;3:221–45.

[46] Grimshaw MN. The audio uncanny valley: sound, fear and the horror game. In: Proceedings 2009. Audio Mostly; 2009, Glasgow, Scotland. p21–6.

[47] Olsen KN . Intensity dynamics and loudness change: a review of methods and perceptual processes. Acoust Aust 2014;42:159–65.

[48] London J. Hearing in time [Internet]. Oxford University Press; 2012. Available from: 10.1093/acprof:oso/9780199744374.001.0001

[49] Hammerschmidt D, Wöllner C, London J, Burger B. Disco time: the relationship between perceived duration and tempo in music. Music Sci (Lond) 2021;4.

[50] Radchenko G, Demareva V, Gromov K, Zayceva I, Rulev A, Zhukova M et al Neural mechanisms of temporal and rhythmic structure processing in non-musicians. Front Neurosci 2023;17:1124038. 37234263 10.3389/fnins.2023.1124038 PMC10206032

[51] Yasuma F, Hayano JI. Respiratory sinus arrhythmia. CHEST 2004;125:683–90. 14769752 10.1378/chest.125.2.683

[52] Kumagai Y, Matsui R, Tanaka T. Music familiarity affects EEG entrainment when little attention is paid. Front Hum Neurosci 2018;12:444. 30459583 10.3389/fnhum.2018.00444 PMC6232314

[53] Huron D . Sweet anticipation [Internet]. The MIT Press eBooks; 2006. Available from: 10.7551/mitpress/6575.001.0001

