# Supplementary material for: Autonomic entrainment to expressive musical phrase structures
Source: Eur Heart J Imaging Methods Pract. 2026 Aug 25;4(3):qyag149. doi: 10.1093/ehjimp/qyag149 (PMC13541111; doi:10.1093/ehjimp/qyag149)
Supplement: Supplementary file 1 [file qyag149_supplementary_data.zip › HFM S2.docx]

**Supplementary Materials S2**

Music stimuli (8 solo-piano performances rendered on a reproducing piano, modulated in tempo and/or loudness to give 30 tracks) and multimodal physiological recordings (ECG-derived RR intervals, respiration, continuous blood pressure) from 126 participants form two parallel processing streams. Beat-level loudness and tempo are segmented by a Bayesian arc-detection algorithm into boundary credence envelopes; physiological signals are filtered, artefact-corrected and converted to Hilbert amplitude envelopes. Both are interpolated onto a shared beat-time grid, and entrainment is quantified as the Earth Mover's Distance between the music boundary envelope and the physiological envelope, with lower values indicating stronger alignment. Participant-specific surrogates provide the null distribution.
