# Supplementary material for: Autonomic entrainment to expressive musical phrase structures
Source: Eur Heart J Imaging Methods Pract. 2026 Aug 25;4(3):qyag149. doi: 10.1093/ehjimp/qyag149 (PMC13541111; doi:10.1093/ehjimp/qyag149)
Supplement: Supplementary file 1 [file qyag149_supplementary_data.zip › HFM S3.docx]

**Supplementary Material S3**

A. The common end point between the HeartFM and BP data allowed each BP segment to be assigned to the corresponding musical track and resampled onto the same beat-time grid used for the music and other physiological signals. Moreover, each participant was asked to squeeze their hand at the beginning of the baseline to also indicate the session start point. Because BP was not synchronised using a hardware trigger or shared device clock, the alignment should be interpreted as track-level temporal alignment rather than millisecond-precise synchronisation. The impact of small timing offsets was in part mitigated by the analysis method which compared smoothed physiological envelopes with phrase-boundary envelopes rather than relying on beat-to-beat point estimates.
B. The algorithm captures this behaviour by modelling segments of the feature time series as quadratic curves, which provide a compact representation of phrase-level expressive shaping. Using Bayesian inference, the algorithm evaluates multiple possible segmentations, weighting them by fit to the data. The initial distributions use prior information from manual annotations.

Rather than enforcing a single, deterministic segmentation of the music, the algorithm adopts a probabilistic framework. It evaluates many possible segmentations of the feature sequence into arcs and assigns each a likelihood based on how well the quadratic model fits the observed data. Using Bayesian inference, these candidate segmentations are weighted by prior information derived from given annotations, allowing the model to favour arc shapes and durations that are musically plausible while still adapting to the specific expressive patterns of each track.

The output of the algorithm is a beat-wise boundary credence profile: a continuous envelope indicating the belief (akin to probability) that a phrase boundary occurs at each beat. Peaks in this envelope correspond to moments where the model infers a high likelihood of a phrase transition. Importantly, this probabilistic representation preserves graded boundary strength rather than imposing binary boundary decisions, reflecting the fact that music phrasing may be ambiguous and vary in perceptual salience.

This boundary credence envelope serves two key purposes in the present analysis. First, it provides a time-resolved representation of music structure that is directly comparable to physiologic signals. Second, by encoding uncertainty and boundary strength, it allows us to test whether stronger or more predictable structural cues are associated with greater physiologic alignment.

C. Other metrics were also tried, such as Euclidean distance, dynamic time warping (DTW) cross-correlation, lagged cross-correlation, and time-frequency coherence. These methods were selected because they have the potential of capturing music-physiology coupling. Cross-correlation and lagged cross-correlation test linear amplitude co-variation at zero or fixed temporal offsets. DTW tests trajectory similarity while allowing differences in local timing. Euclidean distance compares signals on a point-by-point basis. Coherence tests frequency-specific coupling between signals. These analyses did not capture the same details as the distributional analysis through EMD. This led to the interpretation that the primary result is specific to morphology-based boundary-envelope alignment rather than fixed-lag or frequency-specific synchrony. We therefore interpret the EMD results specifically as evidence of boundary-envelope alignment rather than as evidence that all possible synchrony measures identify the same coupling. This distinction is physiologically important because autonomic responses to music may occur with variable latency across participants, tracks, and physiological channels. Metrics requiring fixed point-by-point timing may therefore be less sensitive to phrase-boundary-related physiological changes than EMD, which compares the overall morphology of the music boundary envelope and physiological response envelope.
D. For each participant, track, and music–physiology pairing, we calculated:
   ***uᵢ = P(EMD_surrogate < EMD_original)***
   This was estimated as the proportion of surrogate EMD values lower than the corresponding original value. Since lower EMD indicates stronger alignment, ***uᵢ < 0.5*** indicates stronger original alignment than expected under the participant-specific surrogate null. Under this null, original EMD values should be exchangeable with their surrogate values, giving: ***E[u] = 0.5*** We tested whether: ***ū < 0.5*** and reported Cliff’s delta as the effect size: ***δ = 1 − 2ū***
   Uncertainty was estimated using participant- and track-clustered bootstrap resampling, with the wider interval reported as the conservative estimate. For interpretability, we also reported the median raw EMD shift between the original EMD and the participant-specific surrogate median.
E. Before mixed-effects modelling, surrogate EMD values were summarised to one value per participant, track stimulus, and music–physiology pairing. This prevented the 1000 surrogate iterations from outweighing the original observations. Mean surrogate summaries were used for the main model, and median surrogate summaries were used as a robustness check.
   Three mixed-effects models were fitted. First, an original-versus-surrogate model tested whether EMD differed between original physiological recordings and participant-specific surrogate segments:
   ***EMD ~ condition + feature + signal + track position + familiarity + (1 | participant) + (1 | track stimulus)***
   where condition indicated original versus surrogate, feature indicated loudness versus tempo, and signal indicated respiration, RR intervals, or blood pressure waveform. Track position and familiarity were standardised before modelling.
   Second, an original-only model tested whether entrainment strength differed by music feature, physiological signal, track position, and familiarity within the real physiological recordings:
   ***EMD_original ~ feature + signal + track position + familiarity + (1 | participant) + (1 | track stimulus)***
   Third, an original-minus-surrogate delta model was fitted to provide a direct paired measure of original-versus-surrogate separation:
   ***ΔEMD = EMD_original − EMD_surrogate***
   Negative delta values indicate lower EMD for the original physiological recording than for the participant-specific surrogate, corresponding to stronger alignment during the actual music stimulus. Positive delta values indicate higher original EMD than surrogate EMD. The delta model was specified as:
   ***ΔEMD ~ feature + signal + track position + familiarity + (1 | participant) + (1 | track stimulus)***
