# Supplementary material for: Autonomic entrainment to expressive musical phrase structures
Source: Eur Heart J Imaging Methods Pract. 2026 Aug 25;4(3):qyag149. doi: 10.1093/ehjimp/qyag149 (PMC13541111; doi:10.1093/ehjimp/qyag149)
Supplement: Supplementary file 1 [file qyag149_supplementary_data.zip › HFM S4.docx]

**Supplementary Material S4**

Significant differences observed for tracks between the original and surrogate data indicating that the physiology responds to the music stimulus being listened to. Distinctions between significance of entrainment for the different physiological signals and music feature pairs. L: loudness; T: tempo; R: respiration; RR: RR intervals; BPW: BP waveform, * mark p-values that were statistically significant when comparing the distributions of the original and surrogate results with the Kolmogorov-Smirnov test.
