# Supplementary material for: Autonomic entrainment to expressive musical phrase structures
Source: Eur Heart J Imaging Methods Pract. 2026 Aug 25;4(3):qyag149. doi: 10.1093/ehjimp/qyag149 (PMC13541111; doi:10.1093/ehjimp/qyag149)
Supplement: Supplementary file 1 [file qyag149_supplementary_data.zip › HFM S5.docx]

Supplementary Materials S5

ANOVA results for entrainment outcomes across versions within each piece. Stronger between-version differences are observed for several tempo--physiology pairings.
