# Supplementary material for: Autonomic entrainment to expressive musical phrase structures
Source: Eur Heart J Imaging Methods Pract. 2026 Aug 25;4(3):qyag149. doi: 10.1093/ehjimp/qyag149 (PMC13541111; doi:10.1093/ehjimp/qyag149)
Supplement: Supplementary file 1 [file qyag149_supplementary_data.zip › HFM S6.docx]

**Supplementary Materials S6**

Comparisons of loudness--physiology vs tempo--physiology entrainment outcomes across tracks using the Wilcoxon signed-rank test. Values denote two-sided p-values; lower EMD indicates stronger alignment. Loudness shows stronger entrainment for 27/30 tracks. ⊗ indicates non-significant results or results indicating opposite trend.
