# Supplementary material for: Autonomic entrainment to expressive musical phrase structures
Source: Eur Heart J Imaging Methods Pract. 2026 Aug 25;4(3):qyag149. doi: 10.1093/ehjimp/qyag149 (PMC13541111; doi:10.1093/ehjimp/qyag149)
Supplement: Supplementary file 1 [file qyag149_supplementary_data.zip › HFM Table 1.docx]

Table 1: Pearson correlations between music--physiology EMD and phrase-arc summary statistics. Correlations observed between predictability metrics (arc features and entrainment. L: loudness; T: tempo; P: physiology.

| **Grouping** | | **Statistical test** | |
| --- | --- | --- | --- |
| **EMD Pair** | **Phrase Arc Feature** | **Pearson’s r** | **p-value** |
| L-P | L-T similarity | 0.59 | 8.96E-04 |
| T-P | L-T similarity | 0.93 | 8.49E-14 |
| L-P | Max arc length | 0.87 | 3.05E-08 |
| T-P | Max arc length | 0.91 | 7.99E-12 |
| L-P | Mean arc length | 0.84 | 1.28E-07 |
| T-P | Mean arc length | 0.67 | 5.54E-05 |
| L-P | Std arc length | 0.82 | 3.92E-07 |
| T-P | Std arc length | 0.75 | 2.66E-06 |
